# MicroRNA-208a Silencing Attenuates Doxorubicin Induced Myocyte Apoptosis and Cardiac Dysfunction

**DOI:** 10.1155/2015/597032

**Published:** 2015-06-07

**Authors:** Hasahya Tony, Kunwu Yu, Zeng Qiutang

**Affiliations:** Institute of Cardiology, Union Hospital, Tongji Medical College, Huazhong University of Science and Technology, 1277 Jiefang Avenue, Wuhan 430022, China

## Abstract

*Aims*. GATA4 depletion is a distinct mechanism by which doxorubicin leads to cardiomyocyte apoptosis, and preservation of GATA4 mitigates doxorubicin induced myocyte apoptosis and cardiac dysfunction. We investigated a novel approach of attenuating doxorubicin induced cardiac toxicity by silencing miR-208a, a heart specific microRNA known to target GATA4. *Methods and Results*. Eight-week-old female Balb/C mice were randomly assigned to sham, antagomir, and control groups. Antagomir group were pretreated with miR-208a antagomir 4 days before doxorubicin administration. At day 0, control and antagomir groups received 20 mg/kg of doxorubicin, while sham mice received phosphate buffered solution. Echocardiography was done at day 7, after which animals were sacrificed and hearts harvested and assessed for apoptosis and expression of miR-208a, GATA4, and BCL-2. Doxorubicin significantly upregulated miR-208a, downregulated GATA4, and increased myocyte apoptosis, with resulting decrease in cardiac function. In contrast, therapeutic silencing of miR-208a salvaged GATA4 and BCL-2 and decreased apoptosis, with improvement in cardiac function. *Conclusion*. Doxorubicin upregulates miR-208a and promotes cardiomyocyte apoptosis, while therapeutic silencing of miR-208a attenuates doxorubicin induced myocyte apoptosis with subsequent improvement in cardiac function. These novel results highlight the therapeutic potential of targeting miR-208a to prevent doxorubicin cardiotoxicity.

## 1. Introduction

Doxorubicin, an anthracycline antibiotic, is one of the most effective and widely used drugs in cancer treatment [1]. Its clinical utilization however is limited by its cardiotoxicity, which is dose dependent and cumulative and leads to cardiac dysfunction [2, 3]. Several mechanisms mediating doxorubicin cardiac toxicity have been investigated including cardiomyocyte autophagy and apoptosis [4–7]. However, despite numerous researches delineating the potential mechanisms of doxorubicin induced cardiac toxicity, few effective cardioprotective therapies are available [8]. Given its efficacy and wide use in cancer [8], developing novel therapeutic strategies to mitigate doxorubicin induced cardiac toxicity and dysfunction is of significant importance.

MicroRNAs (miR) are a family of small noncoding RNA molecules that function to regulate gene expression [9–12]. miR-208a is a heart specific microRNA which plays a central role in cardiac stress response [13, 14]. Among the proven targets of miR-208a is GATA4, a cardiac enriched transcription factor whose downregulation following doxorubicin treatment is associated with increased cardiomyocyte apoptosis and heart dysfunction [15–18]. In contrast, increase in GATA4 expression has been shown to decrease cardiomyocyte apoptosis and improve cardiac function following doxorubicin treatment [16, 17]. We thus hypothesized that therapeutic silencing of miR-208a may as well salvage GATA4 and protect against doxorubicin induced cardiac toxicity and dysfunction. Our results showed that antagomir based silencing of miR-208a mitigated doxorubicin induced apoptosis and cardiac dysfunction. These findings, which we believe are novel, open a window for new modalities of preventing the cardiotoxic effects of doxorubicin by targeting miR-208a.

## 2. Materials and Methods

### 2.1. Animal Procedures

All experiments were conducted according to the Guide for the Care and Use of Laboratory Animals, as approved by the Animal Care and Utilization Committee of Union hospital, Tongji Medical College, Huazhong University of Science and Technology, Wuhan, China. The investigation conformed to the Guide for the Care and Use of Laboratory Animals published by the US National Institute of Health (2011).

### 2.2. Animals and Delivery of Antagomir

Adult, female Balb/C mice 8 weeks old were purchased from Tongji Medical College Animal Research Center. The mice were randomly divided into sham (*n* = 11), antagomir (*n* = 8), and control (*n* = 23) groups. Four days before doxorubicin treatment (day −4), antagomir group mice received 50 nmol of miR-208a antagomir each (RiboBio Co., Ltd., Guangzhou, China), in 0.2 ml of normal saline by low pressure tail vein injection, while control and sham group mice received normal saline only. At day 0, control and antagomir group mice received a single intraperitoneal injection of doxorubicin dissolved in phosphate buffered solution (PBS) at a dose of 20 mg/kg, while sham mice received PBS only. Animal survival was recorded over a one-week period.

### 2.3. Echocardiography

To asses if therapeutic silencing of miR-208a attenuates cardiac dysfunction in acute doxorubicin toxicity, animals were lightly anesthetized with pentobarbital sodium (25 mg/kg) on day 7, and two-dimensional transthoracic echocardiography studies performed using Vevo 770 (VisualSonics) ultrasound machine equipped with a 30 MHz cardiac transducer. Cardiac imaging was done in the parasternal short-axis view at the level of the papillary muscles to record M-mode and determine fractional shortening (FS), a measure of contractile function.

### 2.4. Apoptosis Assay

After 7 days, animals were euthanized and hearts were harvested and sectioned. Sections were then fixed in 4% paraformaldehyde and then embedded in OCT compound (BHD, UK) and transversely cut into 5 *μ*m thick cryosections. Apoptosis assay was then done according to previously described protocol [19].

### 2.5. Quantitative Real-Time Polymerase Chain Reaction Analysis

RNA was extracted from homogenized heart tissues using Trizol according to manufacturer's instructions (Invitrogen). For miR-208a detection, we performed RT-PCR using primer sets from RiboBio Co., Ltd., Guangzhou, China, and Taqman probes from Takara Bio Inc. according to manufacturer's instructions. GATA4 and BCL-2 gene expression was analyzed by quantitative RT-PCR using Taqman probes from Takara Bio Inc., with GAPDH as the control. The following primer sets were used.

*GATA4*

 Forward: CGGTTTTCTGGGAAACTGGA
 Reverse: AATTGGATTTGCGGTTGCTC

*BCL-2*

 Forward: TTCGCAGCGATGTCCAGTCAGCT
 Reverse: TGAAGAGTTCTTCCACCACCGT

*GAPDH*

 Forward: GCTGGCGCTGAGTACGTCGTGGAGT
 Reverse: CACAGTCTTCTGGGTGGCAGTGATGG

### 2.6. Statistical Analysis

Statistical analysis was done using SPSS version 20. Groups were compared using independent Student's* t*-test or one-way ANOVA with Turkey or Games-Howell post hoc tests as appropriate. Survival was analyzed using Kaplan-Meier survival curves with Mantel-Cox log-rank test to determine significance. Statistical significance was defined as a *P* value < 0.05 and values are presented as mean ± SEM.

## 3. Results

### 3.1. miR-208a Is Upregulated by Doxorubicin and Its Silencing Attenuates Doxorubicin Induced Cardiomyocyte Apoptosis

**Figure 1:**
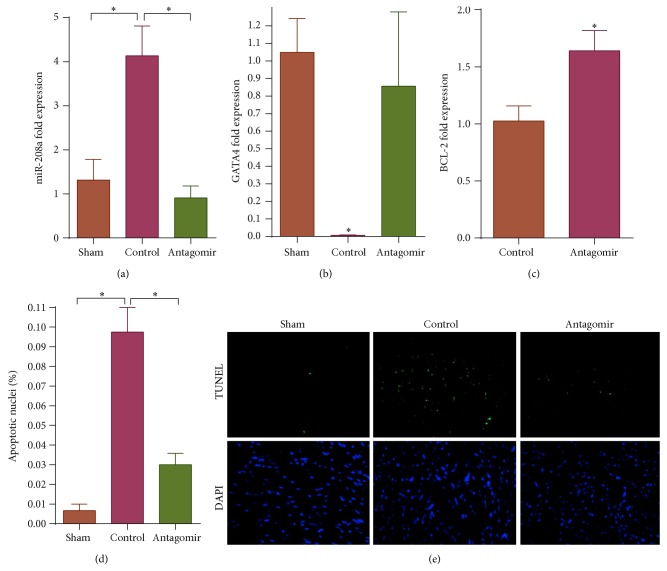
Doxorubicin upregulated miR-208a, downregulated GATA4, and increased apoptosis, while these effects were countered by miR-208a silencing (^*∗*^ *P* < 0.05). (a) Doxorubicin upregulated miR-208a expression, *P* = 0.008, while antagomir pretreatment sufficiently diminished the doxorubicin induced miR-208a upregulation, *P* = 0.003. (b) Doxorubicin decreased cardiac GATA4 expression, *P* = 0.025, while miR-208a antagomir treatment restored GATA4 expression. (c) BCL-2 expression was higher in antagomir pretreated animals than in controls following doxorubicin administration, *P* = 0.033. (d) Doxorubicin significantly increased cardiomyocyte apoptosis in control group, *P* = 0.001, while miR-208a antagomir attenuated doxorubicin induced apoptosis, *P* = 0.002. (e) Representative TUNEL stained images show doxorubicin increased apoptosis in controls compared to sham group mice, while antagomir treated group had significantly less apoptosis compared to controls.

Expression of miR-208a, a heart specific microRNA playing a central role in cardiac stress response and known to target GATA4, was analyzed using quantitative RT-PCR. At 7 days, miR-208a expression was significantly upregulated by doxorubicin treatment. However, therapeutic administration of miR-208a antagomir effectively attenuated doxorubicin induced miR-208a upregulation (Figure 1(a)). Consequently, doxorubicin treatment significantly downregulated GATA4 gene expression, while pretreatment with miR-208a antagomir rescued GATA4 levels (Figure 1(b)). Studies have shown that doxorubicin induced cardiomyocyte apoptosis is in part mediated by GATA4 downregulation [16–18]. GATA4 promotes expression of BCL-2, a known antiapoptotic gene whose upregulation protects cardiomyocytes from various forms of apoptosis [16, 17]. Conversely, GATA4 depletion leads to decrease in BCL-2 with subsequent increase in cellular apoptosis [16, 17]. Thus, having already shown that miR-208a silencing could salvage GATA4, we analyzed BCL-2 gene expression and found that antagomir treated animals had higher BCL-2 levels than controls following doxorubicin treatment (Figure 1(c)).

Given that miR-208a silencing salvaged GATA4, a factor known to decrease doxorubicin induced apoptosis, we analyzed heart sections from the different study groups to see if miR-208a silencing could attenuate doxorubicin induced myocyte apoptosis. Our results showed that doxorubicin significantly increased cardiomyocyte apoptosis, while pretreatment of mice with miR-208a antagomir attenuated doxorubicin induced apoptosis (Figures 1(d) and 1(e)).

### 3.2. Therapeutic Silencing of miR-208a Improves Cardiac Function following Doxorubicin Treatment

**Figure 2:**
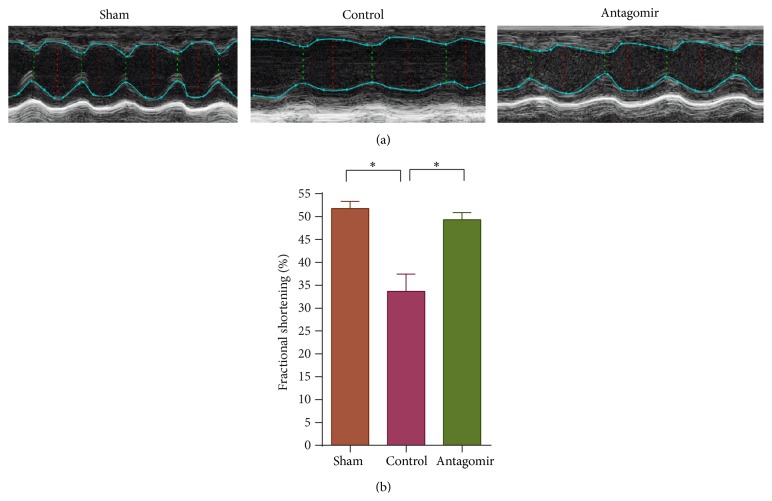
Doxorubicin caused cardiac dysfunction, while antagomir treatment improved cardiac function. (a) Representative images show doxorubicin decreased cardiac function in controls, while antagomir treatment improved cardiac function compared to control. (b) Graph shows doxorubicin decreased cardiac function, *P* = 0.005, while miR-208a antagomir treatment improved cardiac function compared to controls, *P* = 0.011.

To see if miR-208a improves cardiac function, we pretreated mice with 50 nmol of miR-208a antagomir 4 days prior to doxorubicin injection. Two-dimensional transthoracic echocardiography showed that doxorubicin induced cardiac dysfunction, while antagomir treatment attenuated doxorubicin induced cardiac dysfunction as assessed by fractional shortening (Figures 2(a) and 2(b)). Moreover, 20 mg/kg of doxorubicin was lethal in 11 of the 23 (47.8%) control mice, while only 1 of 8 (12.5) antagomir treated mice died during the 7-day follow-up period. However, this difference in mortality did not reach statistical significance (*P* > 0.05) when analyzed using Kaplan-Meier survival curves with Mantel-Cox log-rank test.

## 4. Discussion

GATA4 depletion is a distinct mechanism by which doxorubicin leads to cardiomyocyte apoptosis, and preservation of GATA4 levels has been shown to mitigate doxorubicin induced myocyte apoptosis and cardiac dysfunction [16, 17]. In this study, we report a novel approach of attenuating doxorubicin induced cardiac toxicity by silencing miR-208a, a heart specific microRNA known to target GATA4.

miR-208a is a cardiac specific microRNA which regulates cardiac stress responses [20–23]. It is upregulated in several cardiac diseases including myocardial infarction and dilated cardiomyopathy, in which it is associated with adverse outcomes [20, 23]. Among the proven targets of miR-208a is GATA4, a cardiac enriched transcription factor known to regulate the expression of several cardiac genes including the antiapoptotic gene BCL-2 [15–17]. In the current study, we hypothesized that silencing of miR-208a could salvage GATA4 and attenuate doxorubicin induced myocyte apoptosis. Our results showed that high dose doxorubicin upregulated miR-208a at 7 days, while antagomir was effective in silencing miR-208a upregulation. Therapeutic silencing of miR-208a salvaged GATA4 and decreased myocyte apoptosis in antagomir treated animals compared to controls. During stressful conditions, GATA4 is known to play a role in cardiac adaptive responses and survival, and its genetic deletion induces cardiomyocyte apoptosis and heart dysfunction [24]. One of the mechanisms by which doxorubicin induces myocyte apoptosis and cardiac dysfunction is through depletion of GATA4, and preservation of GATA4 expression has been shown to mitigate doxorubicin induced cardiomyocyte death [16, 17, 24–26]. This protective effect of GATA4 against doxorubicin induced apoptosis is mediated at least in part via upregulation of the antiapoptotic gene BCL-2 [16, 17]. Indeed, in our experiment, BCL-2 was upregulated in antagomir treated group compared to control group. We thus suppose that miR-208a antagomir functions to decreased doxorubicin induced apoptosis at least in part by downregulating miR-208a and hence salvaging GATA4.

In addition, 2D echocardiography showed that miR-208a antagomir treated animals had a higher cardiac function than controls 7 days after doxorubicin treatment. The improved cardiac function was probably due to the reduction in cardiomyocyte apoptosis after antagomir treatment. Moreover, only 1 mouse died in the antagomir treated group, while 6 mice died in the control group, and non died in the sham group during a 7-day follow-up period.

## 5. Conclusion

Taken together, we show that doxorubicin upregulates miR-208a and promotes cardiomyocyte apoptosis, while therapeutic silencing of miR-208a attenuates doxorubicin induced myocyte apoptosis with subsequent improvement in cardiac function. Our results, which we believe are novel, highlight the therapeutic potential of targeting miR-208a to prevent doxorubicin cardiotoxicity.
